# The influence of attention on value integration

**DOI:** 10.3758/s13414-017-1340-7

**Published:** 2017-05-25

**Authors:** Melina A. Kunar, Derrick G. Watson, Konstantinos Tsetsos, Nick Chater

**Affiliations:** 1 Department of Psychology, The University of Warwick, Coventry, CV4 7AL UK; 2 Department of Neurophysiology and Pathophysiology, University Medical Center Hamburg, 20246 Hamburg, Germany; 3 Warwick Business School, The University of Warwick, Coventry, CV4 7AL UK

**Keywords:** Attention, Decision making

## Abstract

People often have to make decisions based on many pieces of information. Previous work has found that people are able to integrate values presented in a rapid serial visual presentation (RSVP) stream to make informed judgements on the overall stream value (Tsetsos et al. *Proceedings of the National Academy of Sciences of the United States of America, 109*(24), 9659–9664, 2012). It is also well known that attentional mechanisms influence how people process information. However, it is unknown how attentional factors impact value judgements of integrated material. The current study is the first of its kind to investigate whether value judgements are influenced by attentional processes when assimilating information. Experiments 1–3 examined whether the attentional salience of an item within an RSVP stream affected judgements of overall stream value. The results showed that the presence of an irrelevant high or low value salient item biased people to judge the stream as having a higher or lower overall mean value, respectively. Experiments 4–7 directly tested Tsetsos et al.’s (*Proceedings of the National Academy of Sciences of the United States of America, 109*(24), 9659–9664, 2012) theory examining whether extreme values in an RSVP stream become over-weighted, thereby capturing attention more than other values in the stream. The results showed that the presence of both a high (Experiments 4, 6 and 7) and a low (Experiment 5) value outlier captures attention leading to less accurate report of subsequent items in the stream. Taken together, the results showed that valuations can be influenced by attentional processes, and can lead to less accurate subjective judgements.

## Introduction

Everyday decisions typically involve taking account of many pieces of information. Making these decisions often requires the evaluation of differing attributes in order to choose the option with the highest overall utility. Previous research in psychology and neuro-economics has examined how people integrate different sources of information and has shown that, in a variety of experimental paradigms, the cognitive system can apply near-optimal strategies and heuristics to make quick and accurate decisions (e.g., Bogacz, Brown, Moehlis, Holmes & Cohen, 2006; Sugrue, Corrado & Newsome, 2005).

Recent work has investigated whether people can make informed overall value judgements by integrating values that appear in rapid succession. Tsetsos, Chater and Usher (2012) developed a *value psychophysics* task in which people viewed two simultaneously presented rapid serial visual presentation (RSVP) streams (one on the left one on the right of the screen), each containing up to 24 numbers. Numbers appeared at a presentation rate of either two or four items per second (across different conditions) and the whole stream was described to participants as reflecting past payments from a slot machine or stock market values. After each trial participants were instructed to choose the stream that they thought had the highest overall value. The results showed that despite the fast presentation rate, participants were surprisingly accurate at amalgamating this information, and chose the stream with the highest value approximately 80% of the time. However, further results showed that people’s value judgements were influenced by certain properties of the stream. First, people appeared to place more weight on the most recently viewed information (i.e., numbers presented towards the end of the stream). Second, people’s judgements seemed biased towards the most extreme values within a stream, and the direction of this bias could be influenced by the framing of the task. For example, when asked to choose the stream with the highest overall value, people chose the stream with the largest standard deviation (SD). However, when asked to reject a stream with the lowest value they also chose the stream with the largest SD. Tsetsos et al. (2012) hypothesised that this was because high SD streams tended to contain more extreme values (both low and high), which participants over-weighted in their judgements. That is, a high SD stream had both very high and very low values, which were compared to the moderate values from the low SD stream. Thus, large numbers from the high SD stream (paired with moderate values from low SD) and moderate numbers from the low SD stream (paired with low values from the high SD stream) were over-weighted, resulting in a choice bias for the high SD stream. When considering which stream was the least valuable, the locally smaller values influenced choice more strongly. This led to the apparently paradoxical finding that the high SD stream was chosen over the low SD stream regardless of whether the task was to choose the stream with the highest or the lowest overall value.

At the same time as making value choices, people also have to sift through multiple sensory inputs to prioritize the most important information, to avoid cognitive overload. Numerous attentional mechanisms that prioritise information have been identified (e.g., Klein, 1988; Treisman & Gelade, 1980; McLeod, Driver & Crisp, 1988; Abrams & Christ, 2003, 2005; Kunar, Flusberg, Horowitz & Wolfe, 2007; Kunar & Humphreys, 2006; Kunar, John & Sweetman, 2014; Kunar, Rich & Wolfe, 2010; Russell & Kunar, 2012; Wolfe et al. 2005; Watson & Kunar, 2010, 2012 see also Wolfe & Horowitz, 2004 and Wolfe & Horowitz, 2017, for a recent review of factors that guide attention). Previous research has suggested that what we pay attention to can be influenced by bottom-up attentional processes (in which inherently salient properties of a stimulus, such as a unique colour, capture our attention, e.g. Treisman & Gelade, 1980), top-down attentional processes (in which cognitive control modulates what it is that we attend to, e.g. Wolfe et al. 1989) and a mixture of both (e.g. Folk et al. 1992). More recent research suggests that attentional control can also be influenced by choice (Kunar, Ariyabandu, & Jami, 2016) and by reward and selection history (see Awh et al., 2012, for a modified framework of attentional control). In the context of the value psychophysics task (Tsetsos et al., 2012), people attended items driven by *top-down processes*, prioritizing the processing of the number that was most relevant to the response mapping (relatively higher values when selecting the overall highest and relatively lower numbers when rejecting the overall lowest stream).

Recent research has begun to investigate whether there is an interaction between attention, bottom-up visual saliency and how we make economic choices. For example, Towal, Mormann and Koch (2013) investigated whether objective properties of saliency are taken into account when participants make subjective value judgements. In their experiment they asked participants to view a grid of four snack foods and choose which they would most like to eat. The visual salience of each of the four stimuli was determined using the Itti-Koch algorithm (Itti, Koch, & Niebur, 1998) and participant’s eye movements to each of the stimuli were recorded. Towal et al. (2013) compared several drift-diffusion models of the data and found that, models that weighted both value and visual salience of the food product outperformed models driven by either value or salience alone. The results suggest that, as well as the subjective value of the food, the bottom-up visual salience of the product also influenced participant’s choices.

Little is known, though, regarding how attention influences overall value judgements when people have to integrate multiple sources of information. Tsetsos et al. (2012) developed a computational model of their data, and showed that their findings could be accounted for by a leaky (decay-based) accumulation model that integrated all samples of value. The model placed higher weights on different values determined by their salience. For example, later samples were weighted more than earlier samples, consistent with experimental findings showing a recency bias (where recently viewed items had a bigger influence on value integration than earlier items). Furthermore, the model accounted for risk-biases and decoy effects shown in the experimental data where higher weights were given to either the high-ranked values or low-ranked values, in the same stream, depending on task framing. However, the model did not consider the role that attentionally salient stimuli play in value integration. That is, although Tsetsos et al. (2012) hypothesised that outlier numbers were over-weighted in a value psychophysics task via top-down strategies, their theory did not consider the impact of bottom-up attentional effects on value judgements. We investigate this in Experiments 1–3 by examining the impact of salient ‘attention-grabbing’ stimuli on value integration. Theoretically, if humans make good decisions that are grounded purely on evidence-based inferences, then the presence of an irrelevant salient item should not affect people’s value judgements. However, if people instead adopt a heuristic that over-weights irrelevant yet salient items, as well as top-down components, we would expect a shift in value preference dependent in the presence of bottom-up factors.

Past attention research has shown that if an item appears in a salient, unique colour it will automatically capture attention (e.g. Treisman & Gelade, 1980, Wolfe, Cave & Franzel, 1989, Duncan & Humphreys, 1989, Wolfe & Horowitz, 2017). Therefore, if attentionally salient items were over-weighted in a stream, judgments of value should be affected by the presence of these items. In Experiments 1–3 we used the value psychophysics paradigm developed by Tsetsos et al. (2012) to test this prediction. In these experiments, participants viewed two RSVP streams of numbers, and indicated which stream had the greatest value. In the critical condition, we highlighted one of the numbers in a salient colour (red in Experiments 1 and 3, green in Experiment 2) which was different to the colours of the rest of the numbers presented in the stream. The highlighted number was either a high value or a low value item.

We determined whether the different coloured item captured attention and subsequently changed people’s judgement of the overall stream value. There are two possible hypotheses. The first predicts, in line with normative decision theory, that because colour has no relevance to the task it should be ignored and thus value judgements would not be influenced by the presence of the uniquely coloured item. We call this the ‘No Attentional Influence’ hypothesis. The second hypothesis predicts that value judgements will be influenced by the presence of a different coloured value in the stream due to its unique and salient bottom-up features. According to this account, if the salient coloured item was of high value, participants would choose this stream as having a higher overall value than they would otherwise. Similarly, if the salient coloured item was of low value, they would choose this stream as having the higher overall value less often. We call this the ‘Attentional Influence’ hypothesis. The results from both experiments showed that value integration was affected by the attentionally salient item, in line with the Attentional Influence hypothesis and concurs with the theory that bottom-up factors play an important role in value judgements.

The second part of our study tests the assumptions of Tsetsos et al.’s (2012) account of their “paradoxical” pattern of judgments, in which participants choose the stream of numbers with the greatest variability whether they were asked to identify the stream with the highest average value, or the stream with the lowest average value. Their theoretical account and computational model showed this result by assuming that, when instructed to choose the highest average value, people will tend to pay more attention to high values, and, conversely, when instructed to choose the lowest average value, they will tend to pay more attention to low values. Thus, the assumption is that effects of task instructions will impact the amount of attention paid to unusually high or low values. Specifically, the more variable stream will tend to be chosen with either set of task instructions, because it has both more unusually high and unusually low values. While this theoretical account provides a simple explanation of the behavioural data, Tsetsos et al. (2012) did not directly measure whether top-down factors were indeed manipulating attention differentially, as the model required. Indeed, their account presupposes, but does not have direct evidence for, the claim that extreme values in general capture attention more than other values.

This was investigated in Experiments 4–7 by examining whether outlier numbers, embedded within the stream, captured attention using a dual-task paradigm, to provide a measure of the degree of attentional engagement with the critical numbers. Typically, in dual-task paradigms if attention is engaged in one task, performance in a secondary task suffers (e.g. Allport, Antonis & Reynolds, 1972; McLeod, 1977; Kunar et al. 2008). Therefore, performance of a secondary task can be used to determine whether extreme values capture more attention than non-extreme values, by the degree to which they deplete attentional resources needed to detect subsequent items in the same stream (e.g. Chun & Potter, 1995).

In Experiments 4–7 we asked participants to detect the presence of a specific target number, while also making a value judgement on the stream. In the critical condition a high-valued (Experiment 4, 6 and 7) or low-valued (Experiment 5) outlier number was presented prior to the target number. We aimed to differentiate between two theoretical options. The first is that outlier numbers do not capture attention. In this case, detection of the target number after viewing an outlier number would be no different to when an outlier number was absent. We call this the ‘No Capture’ account. The second possibility is that presentation of an outlier number would produce a dual-task deficit. Previous research has shown that there is a limit to what we can attend to (e.g., see Marois & Ivanoff, 2005 for a review), and that, when attention is occupied, then the processing of other information in our environment is impaired (e.g. Broadbent, 1958, Pashler, 1994, Rensink, 2002, Simons & Chabris, 1999, Kunar et al. 2008, Kunar & Watson, 2011, 2014, Kunar, Thomas & Watson, 2017). Therefore, if resources were consumed processing the outlier number then there may not be sufficient remaining to process the target item, leading to a detriment in target detection. We call this the ‘Outlier Capture’ account. To preview the results, the data showed that outlier numbers captured attention, impairing detection of subsequent items in the stream. Furthermore, this outlier capture occurred in situations in which observers were asked to make a value judgement to the whole stream (Experiments 4, 5 and 7) or just the target number (Experiment 6) and was greater in earlier lags compared to later ones (Experiment 7). Taken together, the results of Experiments 1–7 suggest that attentional factors modulate the integration of value information and in turn influence people’s judgements.

## Experiment 1

### Method

#### Participants

Twenty participants (13 female) were recruited from the University of Warwick participant pool. Their ages ranged from 18 to 22 years. All participants had normal or corrected to normal visual acuity. We recruited 20 participants per experiment based on the maximum sample size recruited by Tsetsos et al. (2012). A power analysis using the effect size reported in the “equal means” condition in Experiment 2 of Tsetsos et al. (2012) (Cohen’s d = 1.348) showed that the minimum number of participants needed to achieve a power of 0.8 in each experiment was 6.^1^ Therefore, testing 20 participants per experiment should provide ample power to detect significant effects, if present.

#### Stimuli and procedure

Displays were generated, and responses recorded by custom written computer programs running on a PC. Stimuli were two digit numbers that subtended a visual angle of 3.3° by 4.7° at a viewing distance of 57 cm. All stimuli were presented in a light grey colour (RGB values: 180, 180, 180) apart from the salient item, which was presented in red (RGB values: 255, 0, 0). On each trial, participants were presented with a fixation dot (diameter 0.3°) at the centre of the screen for 1000 ms. After this, participants were presented with two streams of 12 numbers each (similar to conditions used by Tsetsos et al. 2012). One stream was presented 4 visual degrees to the left of centre and the other stream was presented 4 visual degrees to the right. Each number in the stream was presented for 250 ms, followed by a blank inter-stimulus interval (ISI) of 50 ms before the next number appeared. In this experiment, we were interested in whether the presentation of a salient (red) item within one of the streams influenced people’s value judgements. Therefore, as we wanted the colour of the crucial item to be the only difference between the two presented streams, unbeknownst to the participants, on each trial, both streams contained the same numbers, presented, however, in the reverse order (from trial to trial the numbers differed). This ensured that both streams had the same means (of approximately 50) and SDs (of approximately 20).^2^

**Fig. 1:**
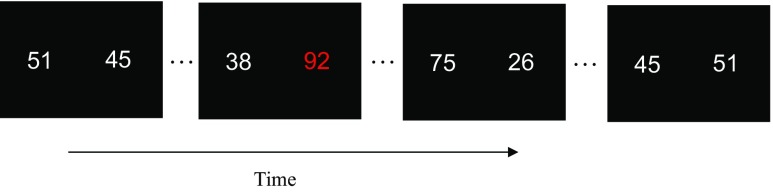
Example display of the Salient Condition in Experiment 1. The Control Condition was identical except that it did not contain an item highlighted in *red*

There were two conditions: a Salient Condition and a Control Condition. In the Salient Condition, 50% of the time the highest value in one of the streams was presented in red, and 50% of the time the lowest value in one of the streams was presented in red. The red item was presented in the left stream for half the trials, and in the right stream for the other half and could appear at any position in either stream. The remaining numbers in each of the streams were presented in light grey. Participants were asked to think of the stream as previous payouts from a slot machine (following the procedure of Tsetsos et al. 2012), and were told that the red item was not informative as to which stream had the highest mean. At the end of each trial participants were asked which of the two streams they thought had the overall highest mean value, by pressing the key ‘z’ if they thought it was the left stream and ‘m’ if they thought it was the right stream. If they were unsure they were asked to guess. No feedback was given. In the Control Condition, all the numbers in both streams were presented in light grey. Therefore, the Control Condition served as a baseline in order to check chance level performance in this task. The presentation of each condition was counterbalanced across participants. The Salient Condition contained 50 trials in which the red item was the highest value in the stream, and 50 trials in which it was the lowest value. The Control Condition contained 50 trials in total. The proportion of times each stream was chosen was recorded and participants performed a short practice block of trials before each condition. Example displays are shown in Fig. 1.

### Results and discussion

Data from one participant were excluded from analysis as they chose the stream with the red item 100% of the time.^3^ As expected in the Control Condition, there was no significant difference in the proportion of times that each stream was chosen, *t*(18) = 1.07, *P* = 0.3, *d =* 0.492 (0.52 versus 0.48, for left and right streams, respectively). Neither did the proportion of times that each stream was chosen differ to that of chance (both *t*s = 1.1, *p*s > 0.29). However, of more interest, in the Salient Condition, the proportion of times that people chose the stream when the highest value number was highlighted in red was greater (0.61) than when the lowest number was highlighted (0.44), *t*(18) = 5.09, *P* < 0.01, *d* = 1.742. Furthermore, the proportion of times that each stream was chosen was higher or lower than chance for the highlighted high and low value streams, respectively. Participants chose the stream with a highlighted high number at a level significantly higher than chance, *t*(18) = 4.04, *P* < 0.01, whereas they chose the stream with a highlighted low number at a level significantly lower than chance, *t*(18) = 3.64, *P* < 0.01.

The results of Experiment 1 are interesting in several ways. Data from our control condition showed that participants were at chance, and chose each stream equally as often when asked to guess which stream had the highest mean. This makes sense as both streams had the same overall mean value. More importantly, the data showed that judgements were influenced by the presence of a red item. In the Salient Condition if the red number was the largest value number, the stream in which it was present was judged to have the highest overall value significantly more often than if the red item was the lowest value item in the stream. The results cannot be accounted for by any difference of the mean or the SD between the two streams because these were identical (Stream 1 was simply Stream 2 presented in the reverse order). The only difference was whether the highest or lowest value item in the stream appeared as the salient red item. This finding shows that value judgements of integrated stimuli can be influenced by attention and illustrate the role of attentional capture in decision-making. In particular, highlighting a number has a positive or negative influence on choosing the associated stream depending on the value (high/low) of the number.

Experiment 2 replicated Experiment 1; however, here the salient singleton was green rather than red. This allowed us to determine that it was the uniqueness of the salient item that was affecting the results rather than being due to, and restricted to, capture by a specific colour.

## Experiment 2

### Method

#### Participants

Twenty participants (18 female) were recruited from the University of Warwick participant pool. The age range of the participants was from 18 to 27 years and all reported normal or corrected to normal visual acuity.

#### Stimuli and procedure

The stimuli and procedure were similar to those of Experiment 1, except that in the Salient Condition, the colour of the salient item was green (RGB values: 0, 255, 0) rather than red.

### Results and discussion

Following the results of Experiment 1, in the Control Condition there was no significant difference in the proportion of times that each stream was chosen, *t*(19) = 0.86, *P* = 0.4, *d =* 1.72 (0.52 versus 0.48, for left and right streams, respectively). However, of more interest, in the Salient Condition the proportion of times that people chose the stream when the highest value number was highlighted in green was greater (0.68) than when the lowest number was highlighted (0.49), *t*(19) = 5.85, *P* < 0.01, *d* = 8.03. Again the results showed that the presence of a salient item affected people’s judgements and that the effect found in Experiment 1 generalised to another colour. People judged the stream to have a higher overall mean more often when the salient item was high in value rather than low, even though there was no objective difference between the means or SDs of the two streams.

One could argue that because the means of both streams were equal in Experiments 1 and 2, people could use the ‘extra’ information provided by the coloured item to influence their decisions. If so the same influence of the salient item should not occur when the means of the stream were different and so there was an objectively correct response to which stream had the highest overall mean. We tested this possibility in Experiment 3, in which participants saw two streams that had different means (60 vs. 40). As in Experiment 1, in the Salient Condition, either the highest or lowest number within one of the streams was highlighted in red. We investigate whether the salient item still influenced people’s judgements when the mean values of each stream were objectively different.

## Experiment 3

### Method

#### Participants

Twenty participants (15 female) were recruited from the University of Warwick participant pool. The age range of the participants was from 19 to 24 years^4^ and all reported normal or corrected to normal visual acuity.

#### Stimuli and procedure

The stimuli and procedure were similar to those of Experiment 1, except that the means of the streams differed. In each trial, one of the streams had a mean of 60 and the other stream had a mean of 40. Both streams had a SD of 20. The stream with the larger mean was presented on the left for half the trials and on the right for the other half. In the Salient Condition, on 50% of the trials, the red item was the largest value in the stream while on the other 50% of the trials it was the smallest value. The red item appeared in the stream that had a mean of 60 half of the time, and the stream that had a mean of 40 for the remaining trials. To ensure equal numbers of trials in each cell, there were 60 trials in which the red item appeared in the stream with a mean of 60 and 60 trials where the red item appeared in the stream with a mean of 40 in the Salient Condition and 60 trials in the Control Condition.

### Results and discussion

**Fig. 2:**
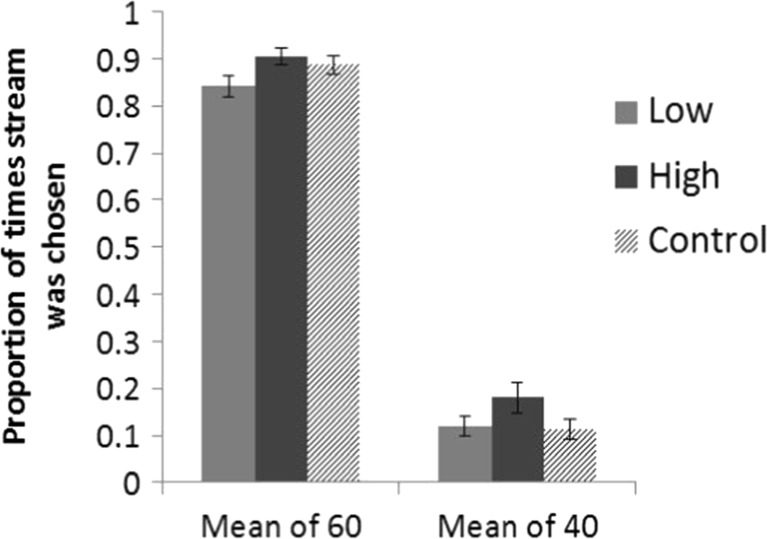
The proportion of times each stream was chosen to have the subjective highest overall mean in the Salient and Control conditions of Experiment 2. *Error bars* Standard error (SE)

Overall, participants accurately identified the stream with the highest mean 86.2% of the time in the Salient condition and 88.7% of the time in the Control condition. The proportion of times each stream was chosen is shown in Fig. 2. For the Salient Condition, the proportion of times each stream was chosen was entered into a 2 × 2 within-subjects ANOVA with factors of Stream Mean (60 or 40) and Red Item (High or Low). The results revealed a significant main effect of Stream Mean, *F*(1, 19) = 347.99, *P* < 0.01, *η*_*p*_^*2*^ = 0.948, participants chose the stream with a mean of 60 more often than the stream that had a mean of 40. There was also a main effect of Red Item, *F*(1, 19) = 15.03, *p* < 0.01, *η*_*p*_^*2*^ = 0.442, participants chose the stream with the high value red item more often than the stream with the low value red item. The Stream Mean × Red Item interaction was not significant, *F* < 1.

The data confirm the findings of Tsetsos et al. (2012) showing that people could successfully integrate a rapid stream of numbers to calculate an overall value. Considering the Control Condition, we see that participants chose the stream with a mean of 60 as having the highest overall value 88.7% of the time. This is significantly above chance, *t*(19) = 18.38, *p* < 0.01, *d* = 0.948, and is consistent with the results of Tsetsos et al. (2012). More importantly, similar to the results of Experiment 1, having either the largest value number or smallest number highlighted in red influenced people’s judgements. Participants were less likely to pick the stream with a mean of 60 as the highest value stream when the red number highlighted a low value, *t*(19) = 3.87, *P* < 0.01, *d* = 0.661.

Furthermore, behaviourally, salient items seemed to bias decisions only when their value was incongruent with the overall value of the stream. Consider the streams with a mean of 60. When the red number was low, people judged the Salient stream as having a lower overall value more frequently compared to the Control condition, *t*(19) = 2.2, *P* < 0.05, *d* = 0.465. However, when the red item was high there was no difference in the proportion of times the Salient stream was picked compared to the Control, *t*(19) = 1.03, *P* = 0.31, *d* = 0.195. Similarly, when the mean of the stream was 40, when the red number was high people judged the Salient stream as having a higher value more frequently compared to the Control condition, t(19) = 3.13, *P* < 0.01, *d* = 0.551. However, when the red item was low there was no difference in the proportion of times the Salient stream was picked compared to the Control, *t* < 1. Overall, when the red item was incongruent with the valuation of the stream mean, it led to less accurate judgements, irrespective of whether stream mean was high or low; conversely, when the red item was congruent with the valuation of the stream mean, it did not improve performance (however, this could be due to a ceiling effect).

Experiments 1–3 showed that embedding a salient item in the stream affected people’s decision making, especially if it was incongruent with other information that was being integrated. These data are important as they add to Tsetsos et al. (2012) model showing that bottom-up attentional factors play a role in value integration. Furthermore, they suggest that if items in the stream become over-weighted they affect people’s value perception. This leads to an interesting question of which items in a stream are weighted higher? Tsetsos et al. (2012) suggested that recently viewed items bias value perception, and Experiments 1–3 here showed that items in a salient colour also bias judgements of value. Tsetsos et al (2012) also suggested that, when asked to pick a stream with the highest or lowest value, streams with a larger SD were chosen more often. They suggested this occurred because streams with a larger SD contained more extreme outlier numbers (e.g. high numbers or low numbers), which captured attention by being over-weighted. We tested this hypothesis directly in Experiments 4–7, in which we examined whether outlier numbers captured more attention in a value psychophysics task than non-outlier numbers. To achieve this we used a dual-task procedure to test two accounts: (1) A No Capture account, which predicts that outlier items do not capture attention; and (2) an Outlier Capture account, which predicts that outlier items will capture attention and disrupt processing of all further items within a stream. In Experiment 4, we investigated whether high outlier numbers capture attention, and in Experiment 5 whether low outlier numbers capture attention. In Experiment 6, we determined whether high value numbers automatically capture attention, or whether they capture attention only when participants are asked to make a judgement of the stream mean. In Experiment 7 we determined whether the dual-task impairment caused by presenting an outlier number differed over lag.

## Experiment 4

### Method

#### Participants

Twenty participants (12 female) were recruited from the University of Warwick participant pool. Their ages ranged from 19 to 23 years. All participants had normal or corrected to normal visual acuity.

#### Stimuli and procedure

The stimuli and procedure were the same as those of Experiment 1, except where reported. In this experiment participants only viewed one stream presented at the centre of the screen. All stimuli were presented in a light grey colour. Numbers were presented at a faster rate of 15 ms/item, with an ISI of 75 ms between each item, matching the presentation rates of other dual-task RSVP studies (e.g., Raymond et al. 1992). Similar to the previous experiments there were 12 numbers in the stream. However, each stream contained two critical values: the first critical value was the outlier in the stream, ranging from 96 to 99 in the High Number trials (outlier present) or a neutral number ranging from 48 to 51 in the Control trials (outlier absent). Each outlier number or neutral number appeared on 12.5% of the total number of trials. This ensured that the outlier numbers did not differ from the neutral numbers in terms of frequency of appearance, but differed only in terms of their magnitude. The second critical value was the target. The target was either the number 19 or 21 (both numbers appearing equally often) and was present on every trial. In all trials, the outlier was presented at position 3 in the stream. To avoid predictability, the target was presented at either position 5 or 11 in the stream with equal probability (Lag 2 or Lag 8, respectively, after the presentation of the outlier). The remaining numbers in the stream varied from 10 to 89, and were generated to either create a desired stream mean of 30 on half the trials, or 70 on the other half of the trials. The average SD for each trial was 16.5. Participants completed 200 trials in a single experimental block (100 High Number trials and 100 Control trials). Participants performed a short practice block of trials before the experiment began.

**Fig. 3:**
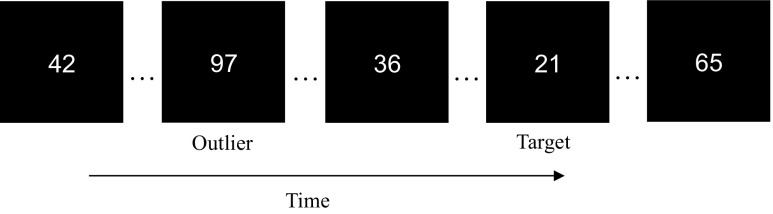
Example display of the rapid serial visual presentation (RSVP) stream in Experiment 3

Participants made two judgements after each stream presentation. First they indicated whether they thought the mean of the stream was higher or lower than 50, by pressing the ‘z’ or ‘m’ key, respectively. This ensured that participants attended to the whole stream, and that they were able to integrate information within the stream to form an accurate judgement. Second, participants responded whether they thought either the number 19 or the number 21 had been presented in the stream by pressing the letters ‘x’ or ‘n’ respectively. They were not asked about the outlier. However, if the outlier automatically captured attention, we would expect to see impairment in target detection when an outlier was present in comparison to when it was absent. Example displays are shown in Fig. 3.

### Results and discussion

Accuracy for reporting whether the mean of the stream was greater or less than 50 was high (89.3%). Trials on which participants did not respond correctly were discounted from further analysis. A two-tailed *t*-test showed that participants were less accurate at detecting the target in the High Number condition (0.66) than in the Control condition (0.69), *t*(19) = 2.22, *P* < 0.05, *d* = 0.172.

Consistent with Tsetsos et al. (2012), people were accurate at calculating the integrated value of the stream. Moreover, this integration occurred at presentation rates of approximately 11 items/s—a rate faster than those that have been presented previously (Tsetsos et al. 2012). The data showed that people’s abilities to integrate multiple sources of information can occur even when information was presented very rapidly.

However, the results showed an overall dual-task deficit. Participants were worse at detecting the target after viewing a high outlier value number compared to having viewed a non-outlier value. If we assume our two tasks to consist of: (1) automatic processing of the outlier number, and (2) detection of the target, the results showed that having an outlier number impaired target detection more (i.e., in the High Number condition) than not having an outlier number in the stream (i.e., the Control condition). The data are difficult to reconcile with a No Capture account, which predicts that there would be no deficit in target detection between the Control and High Number conditions. Instead, the data fit with an Outlier Capture account, which proposes that the presence of a high, outlier number in a stream captures attention, leading to poorer performance overall of the secondary task.

In Experiment 5 we examined whether low outlier numbers also captured attention. If all extreme outliers captured attention we would expect to see a dual-task deficit here. However, if only high value outliers capture attention then no dual-task deficit should be observed.

## Experiment 5

### Method

#### Participants

Twenty participants (14 female) were recruited from the University of Warwick participant pool. Their ages ranged from 19 to 23 years. All participants had normal or corrected to normal visual acuity.

#### Stimuli and procedure

The stimuli and procedure were the same as those of Experiment 4, except that the outlier was a low value outlier ranging from 01 to 04 in the Low Number trials or a neutral number ranging from 48 to 51 in the Control trials. The target was present on every trial and was either the number 79 or 81 (both numbers appearing equally often).^5^ The remaining numbers in the stream varied from 10 to 89, and were generated to either create a desired stream mean of 30 on half the trials or 70 on the other half of the trials. The average SD for each trial was 16.5. Participants completed 200 trials in the experimental block (100 Low Number trials and 100 Control trials).

As in Experiment 4, participants made two responses after each stream presentation. First they indicated whether the mean of the stream was higher or lower than 50 by pressing ‘z’ or ‘m’, respectively, and then whether the number 79 or 81 had been present in the stream by pressing ‘x’ or ‘n’, respectively.

### Results and discussion

Accuracy for determining whether the mean of the stream was above or below 50 was high (86.2%), and incorrect trials were not analysed further. A two-tailed *t*-test showed that participants were less accurate at detecting the target in the Low Number condition (0.65) than in the Control condition (0.69), *t*(19) = 2.96, *P* < 0.01, *d* = 0.386.

Overall, the results were similar to those of Experiment 3. Detection of the secondary target was impaired in the Low Number Outlier condition in comparison to the Control condition. This occurred even though participants were not explicitly asked to attend to the low number. Again, the results rule out a No Capture hypothesis because this would predict that there would be no difference in target detection accuracy between the Control and Low number condition. However, the results concur with an Outlier Capture account, according to which attention was generally depleted after viewing the Low Outlier Number.

Interestingly, in our experiments we found that *both* high and low outlier values captured attention, whereas in Tsetsos et al.’s (2012) work participants only over-weighted items that were congruent with their task instructions (i.e. high value numbers when they were asked to pick the stream with the highest mean, and low value numbers when asked to reject the stream with the lowest mean). The most likely reason why we found overweighting of both high and low numbers in our Experiments 4 and 5 is because of the change in task that we employed. Instead of asking participants to accept or reject a stream based on overall mean (as in the work of Tsetsos et al. 2012), we asked participants to make a judgement as to whether the stream was higher or lower than 50. In doing so, both high and low outliers would be relevant to the task demands, and therefore susceptible to attentional capture as shown. Taken together, the results from both Experiments 4 and 5 showed that embedding an extreme outlier in the stream leads to a dual-task deficit.

One could argue that the reason that the outlier number captured attention was that its value was important in relation to making a judgement about the stream mean. It may be that the outliers receive preferential processing if participants adopt a heuristic of weighing extreme values more when asked to calculate the mean of the stream. In this case, the over-weighting of an outlier would be based on a top-down mechanism applied strategically rather than by the result of automatic attentional capture. We investigate the mechanism behind the over-weighting of outliers in Experiment 6 in which we again ask participants to respond to a target in a stream (which either follows an outlier number or a control); however, we do not ask them to make a judgement of the mean value of the stream. If participants use a top-down heuristic to over-weigh the outlier number when making value judgements, we would now expect to find no dual-task deficit. Alternatively, if high outlier numbers automatically capture attention, we would expect a dual-task deficit when the target appears after a high value item.

## Experiment 6

### Method

#### Participants

Twenty participants (seven female) were recruited from the University of Warwick participant pool. Their ages ranged from 19 to 22 years. All participants had normal or corrected to normal visual acuity.

#### Stimuli and procedure

The stimuli and procedure were similar to those of Experiment 4, except that participants were not asked whether they thought the mean of the stream was higher or lower than 50. Instead they were only asked to respond whether they thought either the number 19 or the number 21 had been presented in the stream by pressing the letters ‘x’ or ‘n’, respectively.

### Results and discussion

Similar to Experiment 4, a two-tailed *t*-test showed that participants were less accurate at detecting the target in the High Number condition (0.68) than in the Control condition (0.72), *t*(19) = 2.88, *P* < 0.01, *d* = 1.808. This occurred even though, in this experiment, participants were not asked to evaluate the stream mean. Therefore the results are inconsistent with the hypothesis that observers adopt a top-down strategy to over-weigh outlier numbers when estimating the mean value of a stream. Instead, the results suggest that outliers automatically capture attention.

Experiments 4–6 found the existence of an overall dual-task deficit when a target number followed an extreme outlier number. Experiment 7 separates target detection across the different lags of the stream (i.e. whether the target appears at Lag 2 or Lag 8 following the outlier or control number). Previous research has shown that detection of a secondary target (T2) is impaired if it appears within 100–500 ms of a primary target (T1, e.g. Raymond, Shapiro & Arnell, 1992, Chun & Potter, 1995). This dual-task deficit is known as the Attentional Blink (AB; Raymond, Shapiro & Arnell, 1992). If we consider the outlier number in our experiments to be acting as a primary target (T1), then we would expect detection of the outlier number (T2) to be worse at Lag 2 than at Lag 8.^6^ Analysing the data across lag in Experiments 4–6 showed that there were no differences in the accuracy of T2 responses between Lag 2 and Lag 8 [*F*(1, 19) = 0.02, *P* = 0.89, η_p_^2^ = 0.001, *F*(1, 19) = 0.09, *p* = 0.76, η_p_^2^ = 0.005, *F*(1, 19) = 0.38, *p* = 0.54, η_p_^2^ = 0.026 for Experiments 4, 5 and 6, respectively). However, the absence of any reliable effects may be a result of insufficient experimental power due to the number of trials used. Therefore, we investigated this in Experiment 7, in which we doubled the trial numbers to allow sufficient data for an analysis across lags to be performed.

## Experiment 7

### Method

#### Participants

Twenty participants (14 female) were recruited from the University of Warwick participant pool. Their ages ranged from 18 to 22 years. All participants had normal or corrected to normal visual acuity.

#### Stimuli and procedure

The stimuli and procedure were similar to those of Experiment 4, except that the number of trials were doubled. Participants completed 400 trials in a single experimental block (200 High Number trials and 200 Control trials).

### Results and discussion

**Fig. 4:**
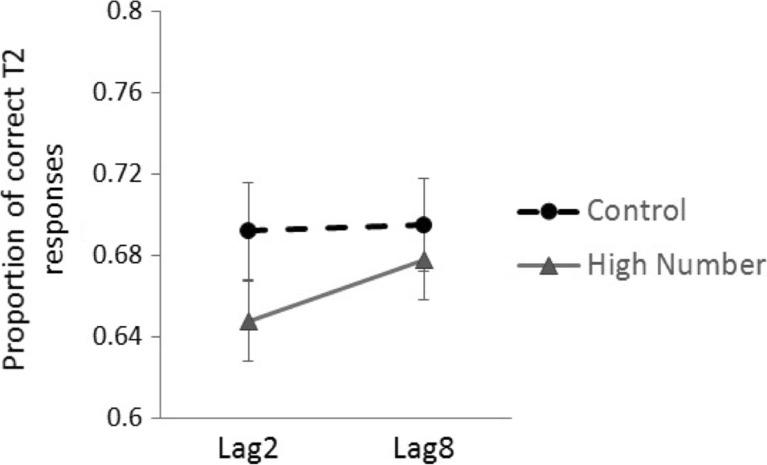
Proportion of correct T2 responses as a function of Lag in Experiment 7. *Error bars* SE

Accuracy for determining whether the mean of the stream was above or below 50 was high (89.4%), and incorrect trials were not analysed further. Figure 4 shows the Proportion of T2 correct responses. The proportion of correct T2 responses were entered into a 2 × 2 ANOVA with factors of Outlier (High Number vs Control) and Lag (Lag 2 vs Lag 8). This revealed a main effect of Outlier, *F*(1, 19) = 8.83, *P* < 0.01, η_p_^2^ = 0.317, participants were more accurate in the Control condition than in the High Number condition. There was a marginal effect of Lag, *F*(1, 19) = 3.74, *P* = 0.07, η_p_^2^ = 0.165, participants were more accurate at Lag 8 compared to Lag 2. The Outlier × Lag interaction was not significant, *F*(1, 19) = 2.06, *p* = 0.17, η_p_^2^ = 0.098. However, planned *t*-tests showed that there was a significant effect of T2 accuracy at Lag 2, where participants were less accurate in the High Number condition compared to the Control, *t*(19) = 2.99, *P <* 0.01, *d =* 0.2, but no effect of accuracy between conditions at Lag 8, *t*(19) = 1.33, *P =* 0.2, *d =* 0.803.

The findings replicate those of Experiments 4–6 showing a dual-task deficit of presenting a high outlier number prior to the target number. Interestingly, separating the data across lag also suggests evidence of an Attentional Blink, with planned *t*-tests showing that accuracy in the High Number condition was lower than in the Control condition at Lag 2, but not at Lag 8. This pattern is consistent with what would be expected from the attentional blink literature, in which a ‘blink’ occurs approximately 100–500 ms following the presentation of a primary target (Raymond et al. 1992). Note that as the Condition × Lag interaction was not significant the data, although suggestive, are not conclusive as to whether an attentional blink occurred. However, importantly, as there was a main effect of Outlier, the data fit with the hypothesis that having a high number embedded in the stream leads to an overall dual-task deficit.

## General discussion

Previous work has shown that when people were shown an RSVP stream containing multiple numbers they were able to rapidly integrate this information to accurately determine the overall value of the stream (Tsetsos et al. 2012). The data were consistent with a model, in which decisions were influenced by the temporal order of items, with more recent values biasing people’s judgements, and by the framing or context of the task (Tsetsos et al. 2012). Additionally, this model encompassed a top-down, response-framing dependent weighting mechanism, that highlighted the larger values when the task was to select the highest stream or the smaller values when the task was to reject the lowest stream. The work in the current paper shows that as well as top-down influences, judgements of integrated value are susceptible to biases by bottom-up factors.

Experiments 1–3 investigated whether adding an irrelevant but unique salient coloured number to the stream, affected people’s judgements in a value psychophysics task. Experiments 1 and 2 had participants make value judgements on two identical streams presented in reversed order, while Experiment 3 had people make value judgements on two streams with different overall means (one a mean of 60 and another a mean of 40). The data showed that if a stream contained a high value coloured number it was more frequently judged to have a higher overall value than if the stream contained a low value coloured item, regardless of the objective stream value. The results showed that embedding an attentionally salient item in the stream affected judgements about the stream’s worth as it captured attention, leading to it being over-weighted. This meant that the salient number biased people’s decision heuristics leading them to interpret the overall value of the stream as higher or as lower than they would have otherwise done.

One reason why the salient item was over-weighted might be due to the isolation effect first reported by von Restorff (1933). The isolation effect occurs when a distinctive item is more likely to be recalled compared to other items that do not have a distinctive feature (Badham & Maylor, 2013, see also Hunt, 1995 & Wallace, 1965). It is hypothesized that the stored representations of the distinctive item have a better quality of encoding due to the uniqueness of the item leading to more elaboration and rehearsal (Schmidt, 1991). According to the Isolation Effect, in Experiments 1–3, the distinct coloured item would have undergone better encoding that would lead to its over-weighting in the stream’s memory representation. Therefore, if participants based their decisions on the availability heuristic (Tversky & Kahneman, 1973), where better recalled information is deemed more important, then their judgements would be influenced by the enhanced stored representations of the coloured item.

Please note that the effect of the salient item might have reflected strategic prioritisation. If participant’s were treating the highlighted number as diagnostic (i.e. a low highlighted number reflected a stream with a lower mean, and a high highlighted number reflected a stream with a higher mean), then they might have formed a strategy to prioritise the salient item in order to make a judgement. In this case, although there was a clear effect of salience, it could be that it was the result of an intentional top-down weighting process.

Experiments 4–7 investigated whether outlier values items in the stream captured attention, leading them to be over-weighted. Tsetsos et al. (2012) suggested that streams with a larger SD were either preferred or rejected depending on whether people were looking for the best or the worst stream, respectively. They proposed that this occurred because streams with a high SD contained extreme outlier values that swayed the decision. We investigated directly whether outlier items captured attention using a dual-task technique. The results showed that both high (Experiments 4, 6 and 7) and low (Experiment 5) outlier values captured attention impairing performance of subsequent target detection. This dual-task deficit was suggestive of an Attentional Blink where the capture provided by the outlier number was worse at Lag 2 than at Lag 8 (Experiment 7). The data were used to test two accounts: a No Capture account, and an Outlier Capture account. It was found that the data were not easily reconciled to a No Capture account, as performance of target detection was worse when an outlier was present compared to when it was absent. Instead, the results favoured an Outlier Capture account in which further processing of subsequent items in the stream were impaired after viewing an outlier item. This suggests that, even when participants were not explicitly told to look for the outlier item, it captured attention, leaving fewer attentional resources available for other items in the stream. The over-weighting of the outlier number also appeared to be automatic/bottom-up as it occurred in conditions where participants were not asked to estimate the value of the stream mean (Experiment 6). These data provide a direct test of Tsetsos et al.’s (2012) model, and concur with their theory that, when integrating, value outlier items would receive a higher top-down attentional weight.

One could argue that, in Experiments 4–7, the fact that the task was to identify a number (e.g., 19 or 21 in Experiments 4, 6, and 7, and 79 or 81 in Experiment 5) encouraged participants to pay attention to each individual value that was presented in the stream. This might have been the reason why the extreme numbers captured attention. If so, then the same effect may not occur if the task was changed so that participants were asked to respond to a non-numeric feature of the target (e.g. look for a red item presented in a multi-coloured stream). It is up to future research to investigate this. However, for now, our results show that, when performing a value-based decision task, extreme outlier numbers capture attention.

The theories surrounding human choice and decision making are complex and sometimes puzzling (Tsetsos et al. 2012, Summerfield & Tsetsos, 2015). Although ideal behaviour is said to be governed by a series of calculated axioms that are essential for optimal decisions (e.g., Von Neumann & Morgenstern, 2007), human behaviour is often shown to be more irrational than this (Summerfield & Tsetsos, 2015), and can be based on sub-optimal decisions (e.g. Kahneman & Tversky, 1979, Stauffer et al. 2015). Our findings add to these data showing that human decisions on value integration were influenced by irrelevant factors of colour. These findings extend the model theorised by Tsetsos et al. (2012) showing that bottom-up factors, as well as top-down factors, play a clear role in value integration. It has been suggested that, in the absence of calculating optimal statistical inferences, observers often employ a range of heuristics when sampling data, some of which are adaptive and provide accurate judgements (Gigerenzer, & Gaissmaier, 2011). Our work shows that, in terms of making judgements and choices of value, participants used a heuristic favouring both bottom-up and top-down attentional cues in decision making.

Taken together, the results have implications for understanding how we evaluate and integrate different sources of information to make overall judgements and decisions. The results run counter to the argument that, in value integration, all items are treated equally. Instead, our results showed that attention plays a vital role in value formation, whether we intend it to or not. People were not asked to attend the salient items in Experiments 1–3 or the outlier values in Experiments 4–7 and yet these attentionally salient items still influenced decisions and had a disproportionate bias on value judgements. Although it might be obvious that salient items captured attention, it is of interest that these attentional factors led to changes in the seemingly unrelated task of decision making. One explanation for this could arise from the idea of reciprocal neural interactions between brain areas involved in attention and emotional valuation (Raymond et al. 2003). Recent research has begun to examine the interaction that attention has on reward and judgements (Anderson et al. 2011, Raymond et al. 2003, Fenske et al. 2004, Raymond, Fenske & Westoby, 2005, Kunar, Watson, Cole & Cox, 2014), suggesting that brain areas involved in these tasks (e.g. the anterior cingulate and the orbitofrontal cortices) are linked by neural circuitry (Fenske et al. 2004, Raymond et al. 2003) and are concurrently activated during attention and evaluation tasks (e.g. Armony & Dolan, 2002; Bush, Luu, & Posner, 2000; Vuilleumier, Armony, Driver, & Dolan, 2001). Knowing this, it follows that manipulating attention may also result in a change in value. Further research is needed to investigate what is occurring on a neural level in the current experiments. Nevertheless, these data have important implications for decision making showing that seemingly irrelevant, yet attentionally salient attributes of a task influence value judgements. Clearly, the way information is presented can affect people’s worth of what was seen, and our data bring together two, previously unlinked, aspects of the human cognitive system, showing that decision making involving the integration of information can be significantly influenced by attentional factors.

## Footnotes

1 Note that this condition in Tsetsos et al. (2012) is analogous to the experimental design here, in the sense that in both cases participants choose between two streams of equal mean and their choice probabilities are compared to chance level (i.e. 50%).

2 Please note that if participants noticed that the streams were identical but reversed we would expect there to be no difference in value judgements across streams, regardless of the presentation of a salient item.

3 We excluded this participant’s data as the pattern of response suggested that the participant did not adhere to the experiment instructions. However, please note that including this data did not affect the overall pattern of results.

4 One participant declined to give their age.

5 The target numbers changed to either 79 or 81 in this experiment as the outlier numbers in the critical condition had changed to lower numbers. This was necessary to meet the mean and variance constraints of the stream.

6 The number in Lag 2 occurs approximately 200 ms after the outlier number, residing in the attentional blink, whereas the number at Lag 8 would appear ~800 ms after the outlier, thereby being outside the attentional blink.
